# Single-Cell TCR Sequencing Reveals the Dynamics of T Cell Repertoire Profiling During *Pneumocystis* Infection

**DOI:** 10.3389/fmicb.2021.637500

**Published:** 2021-04-20

**Authors:** Hu-Qin Yang, Yi-Shan Wang, Kan Zhai, Zhao-Hui Tong

**Affiliations:** Department of Respiratory and Critical Care Medicine, Beijing Institute of Respiratory Medicine, Beijing Chao-Yang Hospital, Capital Medical University, Beijing, China

**Keywords:** *Pneumocystis* pneumonia, T cell receptors, single-cell TCR sequencing, single-cell RNA sequencing, T cell immune repertoire

## Abstract

T cell responses play critical roles in host adaptive immunity against *Pneumocystis*. However, the dynamics and diversity of the T cell immune repertoire in human immunodeficiency virus (HIV)-negative *Pneumocystis* pneumonia (PCP) remains unclear. In this study, single-cell RNA and single-cell T cell receptor (TCR) sequencing were applied to cells sorted from lung tissues of mice infected with *Pneumocystis*. Our findings demonstrated the clonal cells were mainly composed of CD4^+^ T cells in response to *Pneumocystis*, which were marked by highly expressed genes associated with T cell activation. Mice infected with *Pneumocystis* showed reduced TCR diversity in CD4^+^ T cells and increased diversity in CD8^+^ T cells compared with uninfected controls. Furthermore, Th17 cells were mostly clonal CD4^+^ T cells, which exhibited the phenotype of tissue-resident memory-like Th17 cells. In addition, *Pneumocystis*-infected mice showed biased usage of TCRβ VDJ genes. Taken together, we characterized the transcriptome and TCR immune repertoires profiles of expanded T cell clones, which demonstrate a skewed TCR repertoire after *Pneumocystis* infection.

## Introduction

*Pneumocystis* pneumonia (PCP) is a life-threatening complication in human immunodeficiency virus (HIV)-negative immunocompromised patients with more abrupt clinical manifestation, worse prognosis, and higher mortality than HIV patients (Roblot et al., 2002; Roux et al., 2014). The mortality of HIV-negative PCP patients is estimated to range from 20 to 80%, which is higher than in HIV patients (5–15%) (Rilinger et al., 2019; Shoji et al., 2020). The incidence of PCP in HIV-negative patients has continued to increase in recent years (Cilloniz et al., 2019; Lee et al., 2019), highlighting the need to better understand the immune mechanism against *Pneumocystis* to identify new therapeutic targets.

Adaptive immune responses, especially CD4^+^T cells, play a central role in clearing *Pneumocystis* infection and directly influence clinical prognosis (Otieno-Odhiambo et al., 2019). Thus, various studies attempted to elucidate the specific function of CD4^+^ T cells subsets and found that diverse CD4^+^ T cells subsets participate in the immune response against *Pneumocystis* such as Th1 (Garvy et al., 1997), Th17 (Rudner et al., 2007), Treg (McKinley et al., 2006), and Th9 cells (Li et al., 2018). However, those studies were based on whole tissues or bulk populations of cells, which could not account for T-cell heterogeneity at a high resolution, and thus may obscure some important mechanistic mediators.

Recently, the advent of single-cell RNA sequencing (scRNA-seq) has facilitated in dissecting the heterogeneity of immune cells. Furthermore, single-cell T cell receptor (TCR) sequencing (scTCR-seq) can effectively identify clonal cells and characterize their relationships. In infectious diseases, the combination of scTCR-seq and scRNA-seq allows for simultaneous analysis of paired TCR sequences and the transcriptome to track T cell clones (Azizi et al., 2018; Yost et al., 2019). Waickman et al. (2019) characterized the activated and clonally expanded T cells in response to the vaccine of dengue virus and assessed the T cell immune response following vaccination. Wang et al. (2021) delineated the TCR repertoires of coronavirus disease 2019 (COVID-19) patients and observed distinct T cell clonal expansion and skewed VDJ gene usage in COVID-19. Liao et al. (2020) detected the transcriptome and TCR profile of the bronchoalveolar lavage fluid of patients with COVID-19 and found highly clonally expanded CD8^+^ T cells in patients with mild COVID-19.

Li Pira et al. (2001) investigated the characteristics of the TCR repertoire of *Pneumocystis*-specific CD4^+^ T cells in HIV patients using spectratyping; however, changes in TCR repertoires and clonal expansion in multiple T cell subsets in PCP remains unclear.

In this study, we quantitatively tracked the expanded T cell clones and revealed their phenotype during *Pneumocystis* infection using scRNA-seq coupled with scTCR-seq. We comprehensively analyzed the composition and characteristics of clonal T cells, diversity of TCR repertoire, distribution of CDR3 length, and usage patterns of TCRβ VDJ gene segments and combination at different time points post-infection. Our study characterized the dynamics and diversity of TCR profiling across different stages of *Pneumocystis* infection, which contribute to elucidating host adaptive immunity and discovering novel therapeutic targets.

## Materials and Methods

### Mice and Pneumocystis Infection

Adult male C57BL/6J mice and SCID mice aged 6–8 weeks (Vital River Lab Animal Co., Ltd., Beijing, China) were housed under specific pathogen-free conditions at the Beijing Institute of Respiratory Medicine (Beijing, China). C.B-17 SCID mice were inoculated with *P. murina* (American Type Culture Collection, Manassas, VA, United States) as we previously reported (Zhang et al., 2020). For the *Pneumocystis* infection, 1 × 10^6^ *Pneumocystis* cysts contained in 100 μL PBS (Solarbio, Ca^2+^/Mg^2+^-free) were injected through the trachea of each mouse. Control mice were transtracheally injected with 100 μL PBS. To determine the *Pneumocystis* burden, real-time PCR was conducted to determine the copy number of *Pneumocysti*s rRNA from right lung lobes by TaqMan assays. The primers were 5′-AGGTGAAAAGTCGAAAGGGAAAC-3′ and 5′-AAAACCTCTTTTCTTTCACTCAGTAACA-3′. The sequence of probe was 5′-FAM-CCCAGAATAATGAATAAAG-MGBNFQ-3′. Li et al. (2018) and Rong et al. (2019) demonstrated that the *Pneumocystis* burden would increase until the third week, and then decrease in the fourth week after generation of the PCP model. Based on the previously reported *Pneumocystis* burden and our results (Supplementary Figure 1A), we designed this study using mice infected with *Pneumocystis* from 1 to 4 weeks. Thus, research on the mice infected with *Pneumocystis* from 0 to 4 weeks could reflect the full dynamic immune response to *Pneumocystis*.

### Lung Tissue Processing and Cell Sorting

Single cell suspensions were isolated from mouse lungs by collagenase digestion following recently published protocols (Reyfman et al., 2019). Briefly, mice were anesthetized with an overdose of 0.5% pentobarbital, then the lungs were perfused with 1 mL complete 1,640 medium (Solarbio) with 10% FCS (Hyclone) containing collagenase IV (Solarbio) and Dnase I (Sigma) through the trachea, chopped with scissors, and subsequently incubated for 20 min at 37°C with mild agitation. The resulting lung homogenate was passed through a 40-μm filter and resuspended in ACK buffer (BD) for 15 min on ice. Then, the cells were centrifuged at 400 *g* for 6 min and incubated with Percp-Cy5.5 anti-mouse CD45 antibody (BD) and Ghost Dye^TM^ Red 780 (Tonbo) for 15 min on ice. BD FACS ARIA II cell sorter was applied to sort CD45^+^ cells from lung cell suspensions pooled from three mice.

### scRNA-Seq and Data Processing

Single-cell 5′ RNA-Seq libraries were generated using Single Cell 5′ Library and Gel Bead Kit and Chromium Controller (10 × Genomics) following automated cell counting and a sample quality test (Countess^®^ II Automated Cell Counter with AO/PI reagent). After assessing the quality of libraries by Agilent 4200, sequencing libraries were loaded onto an Illumina Novaseq platform. Cell Ranger (v.3.0.2) was applied to demultiplex align reads (mouse GRCm38/mm10 as reference genome) and produce gene-barcode matrices. The Cell Ranger aggr pipeline was used to combine data from all five samples.

### Cell Clustering and Annotation

Quality control and cell clustering were performed using Seurat (version 3.1.2) (Butler et al., 2018). If the genes were less than 200 or more than 4,000 and the mitochondrial genes were more than 20% of total unique molecular identifiers (UMIs), then the cells were excluded from analysis. After performing quality control, gene expression levels were normalized *via* the NormalizeData function and the top 2,000 variable genes were calculated using the FindVariableFeatures function. To reduce the dimensions, the scaled data were used to conduct principal component analysis. Based on the first 20 principal components, the subclusters of T cells were recognized utilizing uniform manifold approximation and projection (UMAP) and t-distributed stochastic neighbor embedding (t-SNE) projections. Clusters were annotated based on the Seurat-based cluster-specific marker gene list and the expression of canonical marker genes, including T cells (Cd3d, Cd3e, and Cd3g), naïve CD4^+^ T cells (Cd4, Sell), effector CD4^+^ T cells (Cd4 and Cd44), naïve CD8^+^ T cells (Cd8a, Sell), effector CD8^+^ T cells (Cd8a and Cd44), γδT cells (Tcrg-C1), double-positive T cells (DPT, Cd4, and Cd8a), NKT cells (Nkg7 and Klrc1), and cycling cells (Mki67 and Stmn1).

We performed Scrublet to exclude cell doublets (Wolock et al., 2019). We calculated the doublet score for each single cell using default parameters and used a cluster-level approach to remove clusters that contained more than 20% doublet cells.

### scTCR-Seq and Analysis

Full-length TCR VDJ segments were generated using the Chromium Single-Cell V(D)J Enrichment kit following the manufacturer’s protocol. The Cell Ranger vdj pipeline was applied to assemble the TCR sequences and identify the CDR3 sequence and TCR genes. Then, the cells were filtered according to the following steps: (1) Cells annotated as T cell clusters in scRNA-seq were kept and (2) Cells that possessed productive TCR α and β chains were incorporated into the analysis. If more than one α or β chain were detected in a cell, then we retained the chain with the highest UMIs (Zheng et al., 2017). We defined the expanded clonal cells as a pair of TCR α and β chains that appeared in at least three cells.

V-J gene combinations were visualized *via* chord diagrams using the circlize package (version 0.4.8). To explore the common and unique V-J and V-D-J gene combinations, we obtained a list of specific V-J and V-D-J gene combinations for all samples, then utilized the Venn Diagram package (version 1.6.20) in R software (version 3.6.1) to display the relationship of those combinations in each sample.

### Differential Expression and Pathway Analyses

The FindMarkers function was used to identify the differentially expressed genes among cell clusters. Genes were considered differentially expressed when the logFC > 0.25, expressed in >25% of cells in the cluster and the adjusted *P* value was < 0.05 based on the Wilcoxon rank sum test. Gene pathway analyses were implemented using the DAVID website^1^.

### Analysis of CD4**^+^** T Cells’ Residency and Migration Scores

Genes of CD4^+^ T cells’ residency and migration were obtained from previous studies (Zhao et al., 2021), then were converted into mouse genes using the Mouse Genome Informatics Database (Supplementary Table 1; Blake et al., 2017). The AddModuleScore function in Seurat was used to calculate the CD4^+^ T cells’ residency and migration scores.

## Results

### scRNA-seq and scTCR-seq Profiling of Lung T Cells

**FIGURE 1:**
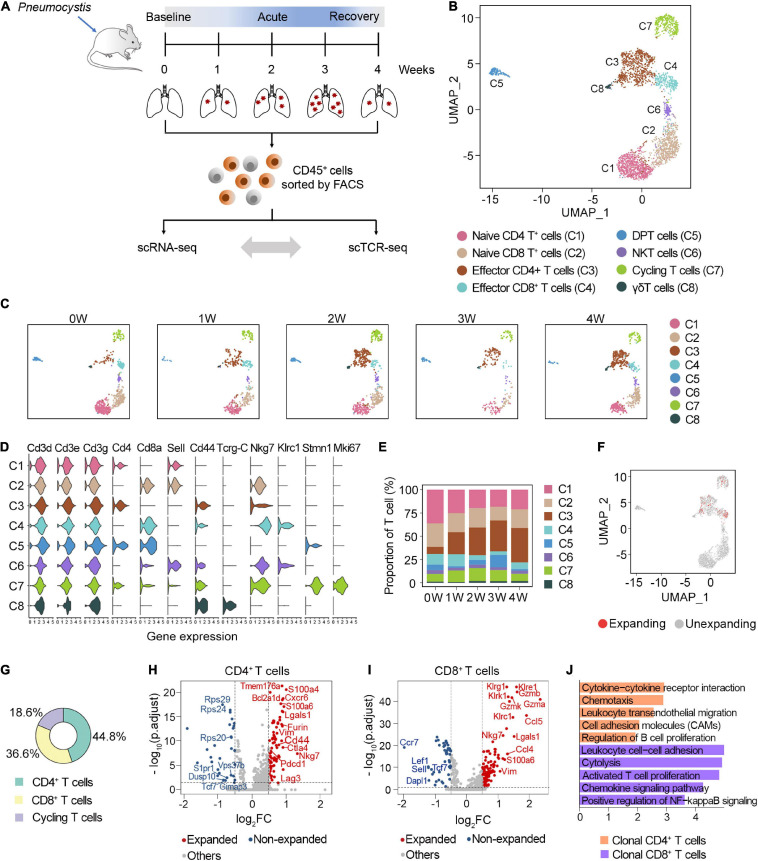
Characterization of clonal T cells by scRNA-seq and scTCR-seq. **(A)** Schematic representation of the experimental design. Immune single cells were isolated from various timepoints during PCP. **(B)** UMAP visualization of T cells. Approximately 2,870 T cells from all samples clustered by UMAP. **(C)** UMAP of T cell clusters from each sample. **(D)** Vlnplot exhibiting the expression of selected markers in each cluster. **(E)** The proportion of T cell subsets at different timepoints. **(F)** UMAP displayed the distribution of clonal T cells. **(G)** The composition of clonal T cells. **(H,I)** Volcano plot showing differentially expressed genes between the expanded and non-expanded cells in CD4^+^ T cells **(H)** and CD8^+^ T cells **(I)**. Each dot indicates one gene; red dots represent a log_2_FC > 0.5, and blue dots depict a log_2_FC < –0.5. Significant genes were selected with adjusted *p*-value < 0.05 based on Wilcoxon rank sum test. **(J)** Gene set enrichment analyses of clonal CD4^+^ T cells and CD8^+^ T cells using DAVID website.

To investigate the dynamics and diversity of the T cell repertoire during PCP, we first performed scRNA-seq and scTCR-seq on lung CD45^+^ cells from mice infected with *Pneumocystis* for 0–4 weeks (Figure 1A). *Pneumocystis* burden continued to increase until 3 weeks post-infection (Supplementary Figure 1A), which was concordant to the previous results of our team. Transcriptional and TCR profiles of single cells were obtained using the 10 × Chromium platform. The sequencing information is shown in Supplementary Table 2. A total of 15,780 T cells were captured in our scTCR-seq analysis from five lung samples, and 61.4% (9,685/15,780) of the T cells detected had full TCR sequences. To simultaneously analyze the transcriptome and immune repertoire of T cells, we only retained the barcodes detected in both scRNA-seq and scTCR-seq. In addition, we excluded the non-productive TCR chains and only kept 2,870 cells with paired α and β chain for subsequent analysis. Finally, unique and productive α chains constituted 47.5% (1,363/2,870) and productive β chains constituted 20.4% of the cells (585/2,870).

### Expansion of Clonal CD4**^+^** T Cells After *Pneumocystis* Infection

T cells were annotated as eight clusters, including naive CD4^+^ T cells (C1), naive CD8^+^ T cells (C2), effector CD4^+^ T cells (C3), effector CD8^+^ T cells (C4), double-positive T cells (C5), NKT cells (C6), cycling T cells (C7), and γδT cells (C8) (Figure 1B). All five samples were well distributed across eight clusters (Figure 1C). The expression of selected marker genes is shown in Figure 1D. We next investigated the dynamic changes of the proportion of each T cell subset during *Pneumocystis* infection. Among these, naive CD4^+^ and CD8^+^ T cells gradually decreased, while effector CD4^+^ T cells significantly increased during *Pneumocystis* infection (Figure 1E). Effector CD4^+^ T cells accounted for only 7.5% of the T cells in the uninfected mice; however, the proportion of effector CD4^+^ T cells significantly increased to 36.8% in the fourth week after *Pneumocystis* infection, supporting previous reports that the role of CD4^+^ T cells in *Pneumocystis* infection was fundamental (Otieno-Odhiambo et al., 2019).

In addition to the T cell frequency, we further analyzed the composition of clonal cells. According to the UMAP projections, the clonal cells were mainly manifested as effector CD4 cells, effector CD8 cells, and dividing cells, while naïve T cells showed minimal clonal expansion (Figure 1F). Among the clonally expanded T cells, 44.8% were CD4^+^ T cells, 36.6% were CD8^+^ T cells, and 18.6% were cycling T cells (Figure 1G). Collectively, these results suggested that clonal CD4^+^ T cells expanded in lung tissues after *Pneumocystis* infection.

We next focused on the potential changes in the transcriptome of clonal T cell populations in PCP. Compared with the unexpanded cells, clonally expanded CD4^+^ T cells were mainly enriched with cytokines, chemokine signaling, and the cell adhesion pathway. Furthermore, clonal CD4^+^ T cells showed the upregulation of genes associated with inhibitory receptors, including Pdcd1, Lag3, and Ctla4 (Figures 1H,J). The increasing expression level of Nkg7, Gzma, Gzmb, and Gzmk in clonal CD8^+^ T cells was related to cytotoxicity (Figures 1I,J). Together, these results indicated the activated, cytotoxic function of clonal T cells in the lung of PCP mice.

### Distinct TCR Immune Repertoire After *Pneumocystis* Infection

**FIGURE 2:**
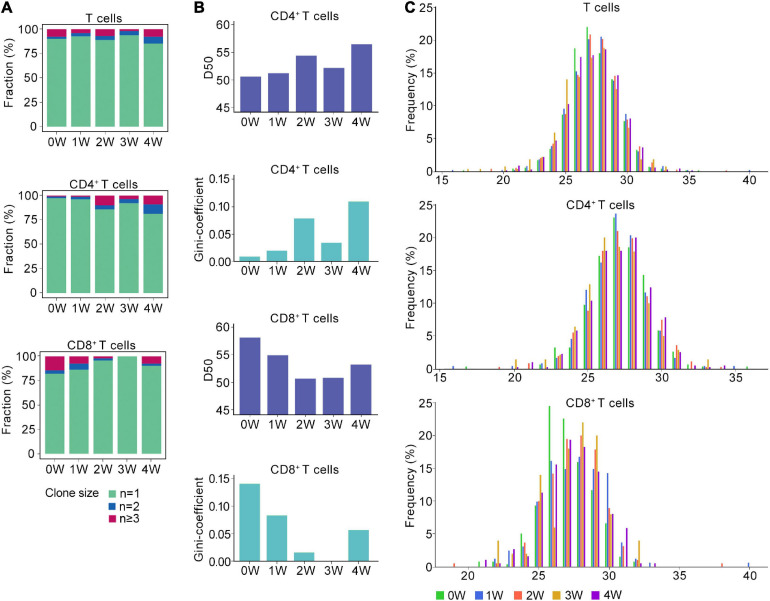
Distinct TCR immune repertoire after *Pneumocystis* infection. **(A)** The proportion of unique and non-unique TCRs expression in T cells (upper), CD4^+^ T cells (middle), and CD8^+^ T cells (lower) across five samples. Unique TCRs (*n* = 1), duplicated TCRs (*n* = 2), and the clonal TCRs (*n* ≥ 3) were labeled with different colors. **(B)** The value of D50 and Gini-coefficient of CD4^+^ and CD8^+^ T cells across samples. **(C)** Distributions of the TCRB CDR3 aa lengths in T cells (upper), CD4^+^ T cells (middle), and CD8^+^ T cells (lower) across all samples.

To evaluate the distribution of clonal TCR, most cells contained unique TCR, while CD4^+^ T cells showed the maximum level of alterations after *Pneumocystis* infection. We found 1.26–10% of the CD4^+^ T cells in the *Pneumocystis* infected lung tissues harbored clonal TCR, which was much higher than the 0.98% clonal TCR in the control mice (Figure 2A and Supplementary Figure 1B). By comparison, the frequency of clonal cells was lower in the CD8^+^ subset and did not markedly change in the total T cells after the *Pneumocystis* infection (Figure 2A and Supplementary Figures 1C,D).

Then, we attempted to characterize the diversity of the TCR repertoire by calculating the D50 value (Kuo et al., 2019). If the value of D50 is closer to 50, then the sample is more diverse. For the CD4^+^ T cells, the D50 of the uninfected sample was 50.7, whereas the values for the *Pneumocystis* infection from weeks 1, 2, 3, and 4 were 51.3, 54.4, 52.2, and 56.5, respectively. For CD8^+^ T cells, the D50 of uninfected samples was 58.2, whereas those of *Pneumocystis* infection from 1 to 4 weeks were 55.0, 50.8, 50.9, and 53.3, respectively (Figure 2B). Furthermore, Gini coefficient was also used to evaluate the diversity and revealed similar results (Figure 2B). Taken together, these results indicated the reduced diversity of CD4^+^ T cells and increased diversity of CD8^+^ T cells in PCP.

TCR β chains are generated by the rearrangement of the V, D, and J genes, which is indicative of a greater diversity than the TCR α chains, and the complementarity determining region 3 (CDR3) is the most variable region of the TCR (Nikolich-Zugich et al., 2004). Therefore, we next analyzed the length of the CDR3 aa in the TCRβ chain. Regardless of the cluster of total T cells, CD4^+^ T cells, or CD8^+^ T cells, the average length of CDR3 aa was 27 bp (range: 16–40 bp), and the length distributions of TCRβ CDR3 were nearly identical in both infected and uninfected tissues (Figure 2C).

### Expansion of Clonal Tissue-Resident Memory-Like Th17 Cells

**FIGURE 3:**
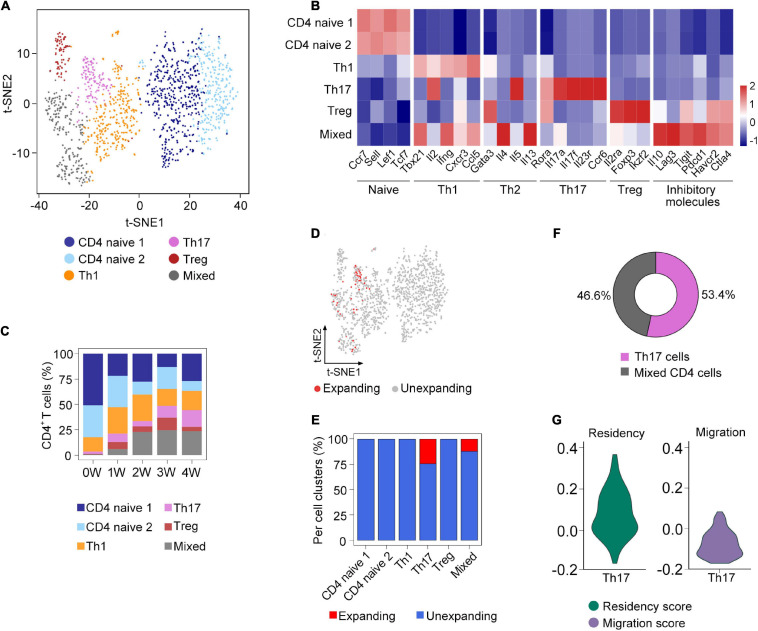
Sub-clustering analysis of clonal CD4^+^ T cells. **(A)** t-SNE plot of the subsets of CD4^+^ T cells from all samples. **(B)** The expression of selected genes in the subsets of CD4^+^ T cells. **(C)** Fraction of each cell subset across five samples. **(D)** Distribution of clonally expanded CD4^+^ T cells on the t-SNE plot. **(E)** Proportion of clonally expanded cells in each CD4^+^ T cells subsets. **(F)** The composition of clonally expanded CD4^+^ T cells. **(G)** Vlnplot showing CD4^+^ T cells’ migration and tissue residency score of all Th17 cells from five samples.

Because CD4^+^ T cells were the most expanded clonal cells after *Pneumocystis* infection, we performed sub-clustering analysis of CD4^+^ T cells from all five samples and identified six subclusters (Figure 3A). According to the gene expression program, two clusters were annotated as naïve CD4 cells, which expressed high levels of Ccr7, Sell, Lef1, and Tcf7. Furthermore, Th1 cell (Tbx21, Il2, Ifng, Cxcr3, and Ccl5), Treg cell (Il2ra, Foxp3, and Ikzf2), and Th17 cell (Rora, Il17a, Il17f, Il23r, and Ccr6) associated gene signatures were identified. Of note, a mixed effector CD4 cluster was observed, which showed upregulated expression of Th1, Th2, and Treg-related genes (Figure 3B). Quantification of cell subsets from scRNA-seq data demonstrated decreased fraction of naïve CD4 cells, while Th1, Th17, and Treg significantly increased after *Pneumocystis* infection. Specifically, the proportion of Th1 cells increased from 14.0% of CD4^+^ T cells at baseline to 26.1% at 2 weeks post-infection. Th17 cells continued to increase from 2.3% of CD4^+^ T cells at baseline to 16.5% at 4 weeks post-infection. Treg cells accounted for only 0.7% of CD4^+^ T cells in the uninfected group, but increased to 12.3% at 3 weeks post-infection, and began to decrease at 4 weeks post-infection (Figure 3C).

To further investigate the composition of clonal CD4^+^ T cells, we compared the clonal expansion of each CD4^+^ T cell subcluster. Interestingly, only Th17 and mixed cells underwent clonal expansion, which accounted for 53.4 and 46.6% of the total clonal CD4^+^ T cells, respectively (Figures 3D–F). Recent studies have demonstrated that Th17 cells could acquire a tissue-resident phenotype in the lungs (Amezcua Vesely et al., 2019). Based on the gene sets obtained from a previous research (Zhao et al., 2021), we calculated the residency and migratory scores of Th17 cells and found that the Th17 cells in our study have a relatively higher residency score (Figure 3G), suggesting that Th17 cells might exhibit the phenotype of tissue-resident memory-like Th17 cells (Trm-like 17 cells).

### The Altered Usage Patterns of TCRβ VDJ Gene Segments and Combination After *Pneumocystis* Infection

**FIGURE 4:**
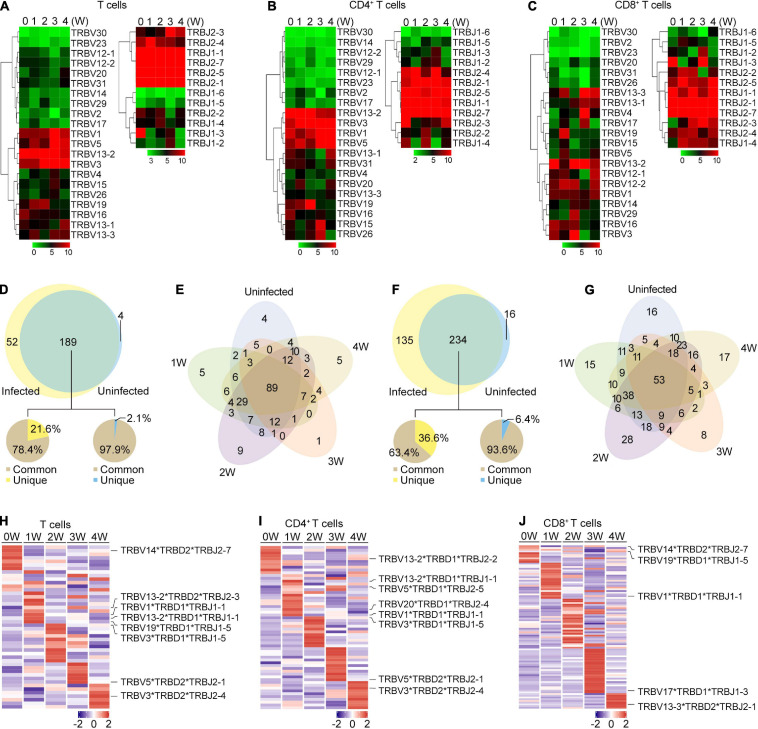
TRB gene usage in *Pneumocystis* infected and uninfected mice. **(A–C)** Heat maps of V and J gene usage for TRB in T cells **(A)**, CD4^+^ T cells **(B)**, and CD8^+^ T cell **(C)** at different timepoints. **(D)** Venn diagram showing the common and unique V-J gene pairs of *Pneumocystis*-infected mice and uninfected controls. Pie charts showing the proportion of cells that harbored common and unique V-J pairs in *Pneumocystis*-infected mice (left) and uninfected controls (right). **(E)** Venn diagram showing the common and unique V-J gene pairs of mice infected with *Pneumocystis* from 0 to 4 weeks. **(F)** Common and unique V-D-J gene pairs of *Pneumocystis*-infected mice and uninfected controls. Pie charts showing the proportion of cells that harbored common and unique V-D-J pairs in *Pneumocystis*-infected mice (left) and uninfected controls (right). **(G)** Venn diagram showing the common and unique V-D-J gene pairs of mice infected with *Pneumocystis* from 0 to 4 weeks. **(H–J)** Heat maps showing the top 10 V-D-J gene combinations that were most frequently used in T cells **(H)**, CD4^+^ T cells **(I)**, and CD8^+^ T cells **(J)**, of which the most frequently used combinations are shown in labels.

To gain insights into the preference of TCRβ VDJ gene segments, a total of 21 V gene and 12 J gene segments were detected in TCRβ from five samples. As shown in the heat map, the usage patterns of the V and J gene segments were similar between the different time points of the PCP and healthy control groups (Figures 4A–C and Supplementary Table 3). For the total T cells, the most frequent V gene segments in *Pneumocystis* infected in the 0–4 W groups were TRBV13-2 (14.3%), TRBV13-2 (9.8%), TRBV3 (11.9%), TRBV1 (11.1%), and TRBV3 (10.7%), respectively (Supplementary Figure 1E). For CD4 cells, the most frequent V gene segments in the 0–4 W groups were TRBV13-2 (10.4%), TRBV13-2 (10.4%), TRBV3 (14.1%), TRBV3/TRBV1 (13.6%), and TRBV3 (12.4%), respectively (Supplementary Figure 1F). For CD8 cells, the most frequent V gene segments in the 0–4 W groups were TRBV13-2 (19.5%), TRBV19 (9.3%), TRBV13-2 (11.6%), TRBV13-2/TRBV13-3/TRBV4 (14%), and TRBV13-3 (9.1%), respectively (Supplementary Figure 1G). TRBJ2-7 accounted for the largest number of TRBJ in the total T cells and CD8 cells for each sample, except for TRBJ1-1 in CD4 cells at 3 W after infection (Supplementary Figures 1E–G). TRBD gene usage was also examined, and TRBD1 genes were used most frequently across all samples. Therefore, alterations in the TCRβ VDJ gene segments mainly occurred in the V gene in PCP.

We also examined the make-up of V-J and V-D-J gene combinations for TCRβ and identified 245 distinct V-J gene combinations and 385 distinct V-D-J gene combinations (Supplementary Tables 4, 5). To identify the disease-specific TRB V-J and V-D-J gene combinations, we quantified the common and unique V-J and V-D-J gene combinations between infected and uninfected samples (Supplementary Table 6). The results showed that of all the 241 V-J combinations in *Pneumocystis*-infected mice, 189 (78.4%) were shared with uninfected controls, and 52 (21.6%) were unique (Figure 4D). The common and unique V-J combinations between each sample is shown in Figure 4E, which suggest that unique V-J combinations existed at every time point. Greater variation was observed in the V-D-J gene combinations, of all the 369 V-D-J combinations in *Pneumocystis*-infected mice, 234 (63.4%) were shared with uninfected controls and 135 (36.6%) were unique (Figure 4F). Unique V-D-J combinations were also obtained in all samples, ranging from eight to 28 (Figure 4G). The frequency of all V-J and V-D-J combinations in T, CD4^+^ T and CD8^+^ T cells were visualized *via* circos plots and heat maps, respectively (Supplementary Figures 2, 3). The most frequently used V-J and V-D-J combinations are listed in Supplementary Table 7. In addition, we constructed heat maps of top 10 V-D-J gene combinations that were most frequently used in T, CD4^+^ T, and CD8^+^ T cells, which showed distinct patterns of V-D-J gene combinations for TCRβ at each time point (Figures 4H–J). These results demonstrated that the distributions of the V-J and V-D-J combinations were significantly shaped by *Pneumocystis* infection.

## Discussion

An increasing number of studies have attempted to elucidate the mechanism of T cells in controlling *Pneumocystis* infection, which indicate that *Pneumocystis* infection activates helper T cells and promotes cytokine production (Otieno-Odhiambo et al., 2019). However, the modulation of TCR immune repertoires in PCP remains unclear. Concordant with previous studies, we revealed that *Pneumocystis* induced the enrichment of effector CD4^+^ T cells. By quantifying the clonal cells, we found that the highly expanded clones were mainly composed of CD4^+^ T cells. In addition, *Pneumocystis* infection induced the proliferation of clonal CD4^+^ T cells, and the expansion degree of clonal CD4^+^ T cells was greater than that of CD8^+^ T cells. In combination with single-cell transcriptomics, we discovered that the clonal CD4^+^ T cells expressed genes were associated with cytokine, chemokine signaling, and cell adhesion pathway, whereas CD8 cells mainly expressed cytotoxic effector genes. The clonally expanded CD4^+^ T cells highly expressed immune checkpoint molecules, including Pdcd1, Lag3, and Ctla4, which confirmed our recent findings of elevated expression of PD-1/PD-L1 in PCP. Zhang et al. (2020) also found anti-PD-1 antibody could promote the clearance of *Pneumocystis*, which might be due to the inhibition of expansion in these clonal CD4 cells with highly expressed inhibitory checkpoints.

In addition, we discovered the decreased diversity of CD4^+^ T cells and increased diversity of CD8^+^ T cells in PCP, which suggested differences in the roles of CD4^+^ and CD8^+^ T cells in response to *Pneumocystis*. Prior studies have shown that CD4^+^ T cell-mediated response is absolutely pivotal in controlling *Pneumocystis* infection (Kelly and Shellito, 2010), as we observed clonal expansion and decreased diversity of CD4^+^ T cells. However, the role of CD8^+^ T cells in PCP remains controversial, with researchers describing both protective and detrimental functions (McAllister et al., 2004; Gigliotti et al., 2006; Zhang et al., 2019). In our study, as shown in Figure 2A, little clonal expansion was observed in CD8^+^ T cells after the *Pneumocystis* challenge.

The sub-clustering analysis of CD4^+^ T cells revealed that *Pneumocystis* induced the accumulation of Th17 cells, and clonally CD4^+^ T cells were mostly represented as Th17 cells. Concordant to our results, previous studies have demonstrated that neutralizing IL-17A would lead to a significant increase in *Pneumocystis* burden in the lungs (Rudner et al., 2007), suggesting that Th17 response play a critical role in *Pneumocystis* clearance. In addition, we observed that Th17 cells might resemble the phenotype of Trm17 cells. Recent studies have shown that Trm17 cells could be induced in the lungs following bacterial or fungi infection. Trm17 cells were thought to play a crucial role in protecting from *Klebsiella* pneumonia infection (Amezcua Vesely et al., 2019). These results indicated the potential role of Trm17 cells in *Pneumocystis* clearance; however, its specific function in PCP requires further investigation.

Although the distribution of the CDR3 aa lengths remained similar after *Pneumocystis* infection, we found that the frequencies of some V and J gene segments significantly increased at different time points upon *Pneumocystis* infection, as well as the preferential usage of V-J and V-D-J gene combinations. Furthermore, through investigating the common and unique V-J and V-D-J combinations between all samples, we found that the unique combinations significantly increased in PCP compared to the control group. However, the function of these unique V-J and V-D-J combinations remains unclear.

While this work comprehensively delineates changes in clonal T cells and V(D)J gene usage for PCP, several key matters require validation. First, we only obtained data from mice infected with *Pneumocystis* at different timepoints, which is not sufficient to reflect the pathogenesis of human PCP. Second, readily visible clonal expansion was observed in Th17 cells that had a phenotype resembling Trm17 cells after *Pneumocystis* infection. The number and function for Trm17 cells in PCP need to be further explored. V and J gene segments and combinations that significantly increased in PCP in this study also need validation. Finally, the limited sample size of our study is another limitation, therefore, the results of this study require validation using a larger PCP patient cohort.

In conclusion, investigations on T cell immunity have always been an important subject in PCP. Due to the lack of studies on the TCR repertoire of PCP, we performed scTCR-seq and scRNA-seq to comprehensively reveal the reconstructed TCR repertoire during *Pneumocystis* infection at the single-cell resolution and to track the clonal cells uniting TCR clonotype and transcriptome phenotype, thereby providing novel insights to improve our understanding of adaptive immune response in PCP.

## Data Availability Statement

The sequence data in this study were deposited in the Gene Expression Omnibus (GEO). The accession number of scTCR-seq data is GSE156843 and that of scRNA-seq is GSE157627.

## Ethics Statement

The animal study was reviewed and approved by Capital Medical University Animal Care and Use Committee.

## Author Contributions

H-QY conducted the experiments and drafted the manuscript. H-QY and Y-SW performed the data analysis. KZ and Z-HT designed the experiments, supervised the study, and revised the manuscript. All authors contributed to the article and approved the submitted version.

## Conflict of Interest

The authors declare that the research was conducted in the absence of any commercial or financial relationships that could be construed as a potential conflict of interest.

## Footnotes

1 https://david.ncifcrf.gov

## Supplementary Material

Supplementary Figure 1 *Pneumocystis* burden, TCR clones, and V/J gene segments usage at each time point. **(A)** Changes in *Pneumocystis* burden in mice 0–4 weeks post-infection. **(B–D)** The proportion of clonal cells (*n* ≥ 3) in CD4^+^ T cells **(B)**, T cells **(C)**, and CD8^+^ T cells **(D)** at each time point. **(E–G)** The fraction of V and J gene frequency for TCRβ in T cells **(E)**, CD4^+^ T cells **(F)**, and CD8^+^ T cells **(G)** visualized using bar plots.

Supplementary Figure 2 TRB V-J gene combinations in five samples. V-J gene combinations were labeled when the frequency was more than 1%.

Supplementary Figure 3 Usage frequency of V-D-J gene combinations at each time point. **(A–C)** Heat maps showing usage frequency of V-D-J gene combinations in T cells **(A)**, CD4^+^ T cells **(B)**, and CD8^+^ T cells **(C)**.

Supplementary Table 1 Gene list. This table provides the gene information of residency and migratory scores of CD4^+^ T cells.

Supplementary Table 2 Sequencing information of scTCR-seq. This table provides the information about scTCR-seq of all five samples, which were summarized by CellRanger.

Supplementary Table 3 Frequency of V, D, and J gene segments in T, CD4^+^ T, and CD8^+^ T cells in each sample.

Supplementary Table 4 Frequency of TRB V-J pairs for T, CD4^+^ T, and CD8^+^ T cells in each sample.

Supplementary Table 5 Frequency of TRB V-D-J combinations in T, CD4^+^ T, and CD8^+^ T cells in each sample.

Supplementary Table 6 A list of common and unique V-J combinations in Figure 4E and V-D-J combinations in Figure 4G.

Supplementary Table 7 The most frequently used V-J and V-D-J combinations for T, CD4^+^ T and CD8^+^ T cells across all samples.
