# Supplementary figures and images for: Single-Cell TCR Sequencing Reveals the Dynamics of T Cell Repertoire Profiling During Pneumocystis Infection
Source: Front Microbiol. 2021 Apr 20;12:637500. doi: 10.3389/fmicb.2021.637500 (PMC8093776; doi:10.3389/fmicb.2021.637500)

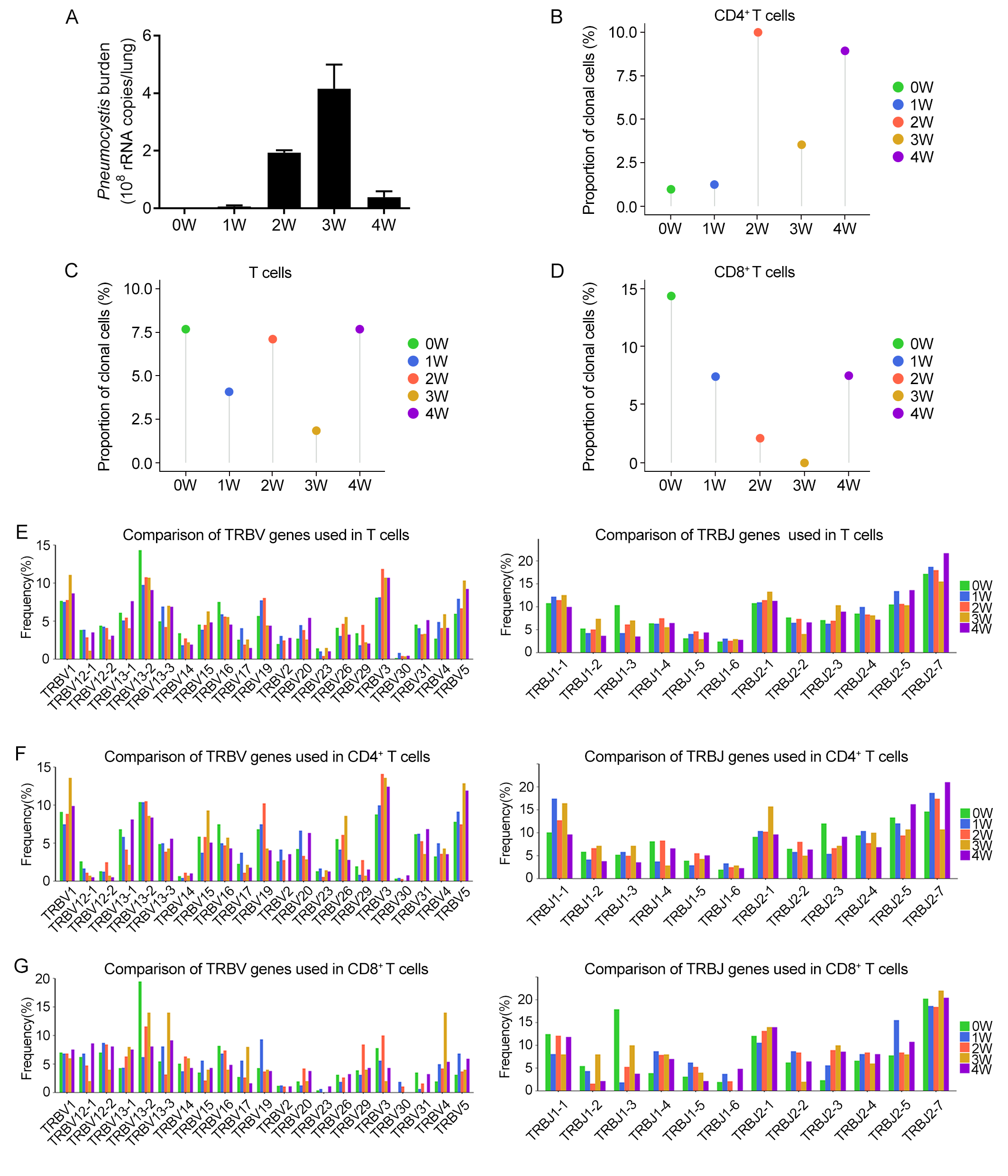

Supplement: Supplementary Figure 1 — Pneumocystis burden, TCR clones, and V/J gene segments usage at each time point. (A) Changes in Pneumocystis burden in mice 0–4 weeks post-infection. (B–D) The proportion of clonal cells (n ≥ 3) in CD4+ T cells (B), T cells (C), and CD8+ T cells (D) at each time point. (E–G) The fraction of V and J gene frequency for TCRβ in T cells (E), CD4+ T cells (F), and CD8+ T cells (G) visualized using bar plots. [file Image_1.TIF]

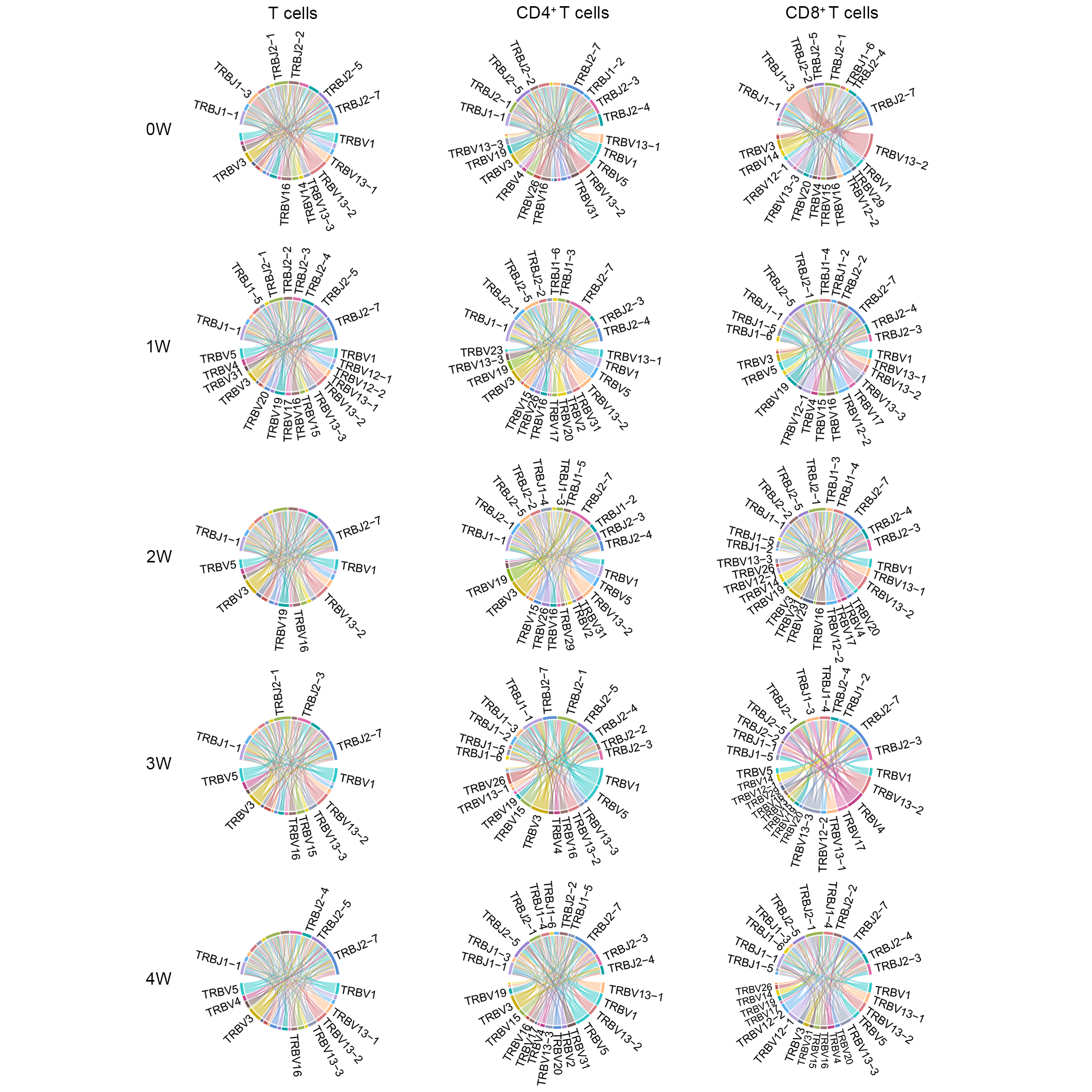

Supplement: Supplementary Figure 2 — TRB V-J gene combinations in five samples. V-J gene combinations were labeled when the frequency was more than 1%. [file Image_2.TIF]

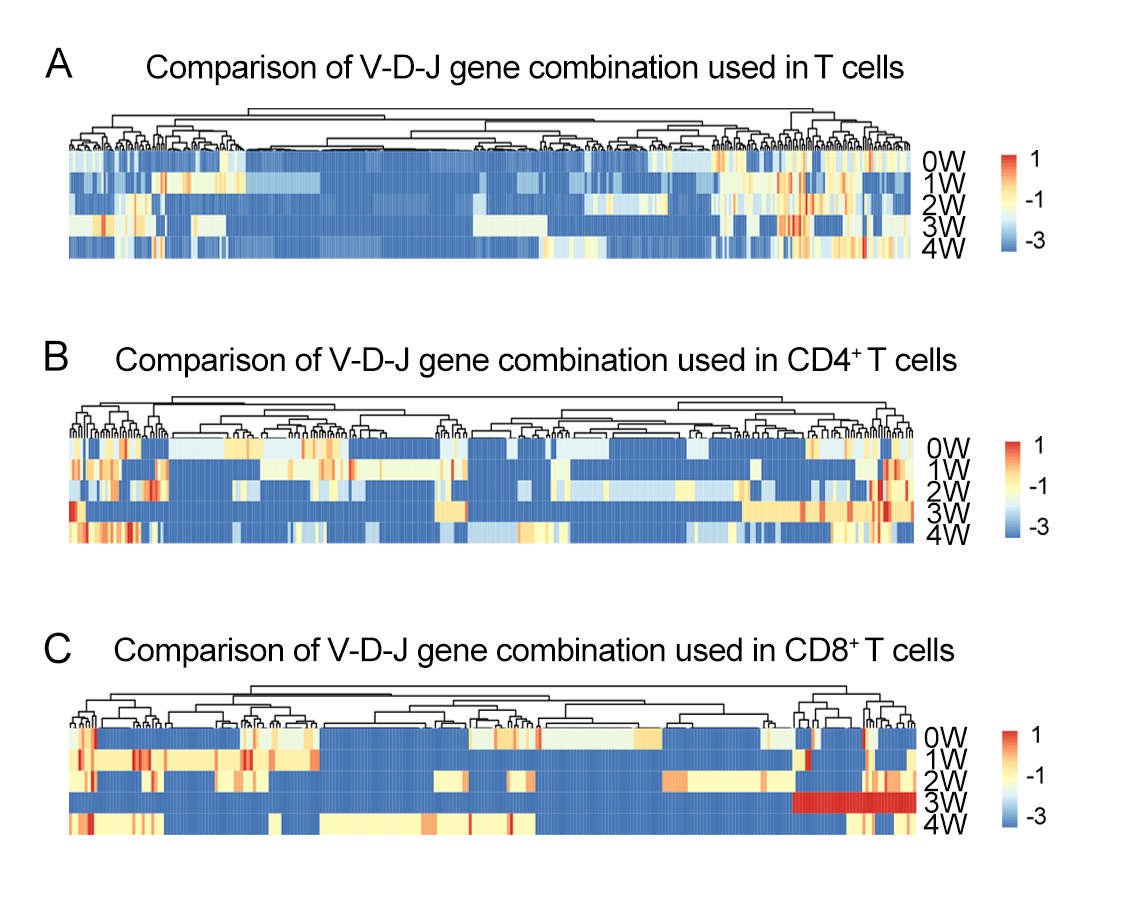

Supplement: Supplementary Figure 3 — Usage frequency of V-D-J gene combinations at each time point. (A–C) Heat maps showing usage frequency of V-D-J gene combinations in T cells (A), CD4+ T cells (B), and CD8+ T cells (C). [file Image_3.TIF]
